# Measuring adherence to antiretroviral therapy in northern Tanzania: feasibility and acceptability of the Medication Event Monitoring System

**DOI:** 10.1186/1471-2458-11-92

**Published:** 2011-02-09

**Authors:** Ramsey A Lyimo, Jossy van den Boogaard, Elizabeth Msoka, Harm J Hospers, Andre van der Ven, Declare Mushi, Marijn de Bruin

**Affiliations:** 1 Kilimanjaro Clinical Research Institute/Kilimanjaro Christian Medical Center P.O.Box 2236, Moshi, Tanzania; 2 Radboud University Nijmegen Medical Center, P.O. Box 9101, 6500 HB, Nijmegen, the Netherlands; 3 Maastricht University, Faculty of Psychology and Neuroscience, the Netherlands; 4 Kilimanjaro Christian Medical College, Community Health Department, P.O.Box 2240, Moshi, Tanzania; 5 Wageningen University, Communication Science, the Netherlands

## Abstract

**Background:**

An often-used tool to measure adherence to antiretroviral therapy (ART) is the Medication Event Monitoring System (MEMS), an electronic pill-cap that registers date and time of pill-bottle openings. Despite its strengths, MEMS-data can be compromised by inaccurate use and acceptability problems due to its design. These barriers remain, however, to be investigated in resource-limited settings. We evaluated the feasibility and acceptability of using MEMS-caps to monitor adherence among HIV-infected patients attending a rural clinic in Tanzania's Kilimanjaro Region.

**Methods:**

Eligible patients were approached and asked to use the MEMS-caps for three consecutive months. Thereafter, qualitative, in-depth interviews about the use of MEMS were conducted with the patients. MEMS-data were used to corroborate the interview results.

**Results:**

Twenty-three of the 24 patients approached agreed to participate. Apart from MEMS-use on travel occasions, patients reported no barriers regarding MEMS-use. Unexpectedly, the MEMS-bottle design reduced the patients' fear for HIV-status disclosure. Patients indicated that having their behavior monitored motivated them to adhere better. MEMS-data showed that most patients had high levels of adherence and there were no bottle-openings that could not be accounted for by medication intake. Non-adherence in the days prior to clinic visits was common and due to the clinic dispensing too few pills.

**Conclusion:**

MEMS-bottle use was readily accepted by patients. Although the MEMS-bottle was used accurately by most patients, patients need to be more explicitly instructed to continue MEMS-use when travelling. Even HIV-clinics with sufficient staff and free medication may impose structural adherence barriers by supplying an insufficient amount of pills.

## Background

The UNAIDS report of 2009 estimated the global HIV prevalence at 33.4 million people, of whom 67% are living in Sub-Saharan Africa. Two million people died of AIDS in 2008 and 70% of these deaths occurred in Sub-Saharan African countries [1]. According to the World Health Organization (WHO), the antiretroviral therapy (ART) coverage was 44% in Sub-Saharan Africa by the end of 2008 [2]. In Tanzania, the estimated adult HIV prevalence was 5.7% in 2009 (equivalent to 1.4 million people), and 96,000 died of AIDS in 2008 [3]. By March 2009, 55.2% of the 425,725 Tanzanian children and adults in need of ART were receiving it [4].

Scaling up the availability of ART is crucial for reducing mortality and HIV-transmission [5]. ART offers an opportunity to prolong lives of people living with HIV/AIDS (PLWHA) and revive societies that have been affected by the epidemic. While access to ART is vital, it is equally important to ensure that patients adhere to the prescribed regimen. High levels of adherence (>90-95%) are required for long-term viral suppression to delay progression to AIDS [6]. Suboptimal adherence to ART increases the risk of drug resistance development. The emergence of drug resistance is a serious concern, especially in settings where the options for second-line treatment are limited [7]. Therefore, monitoring the patients' adherence to ART is a requirement for adequate HIV care provision and research.

The accurate measurement of adherence is a challenge and at present there is no gold standard [8,9]. A tool that has been used extensively in developed countries is the Medication Event Monitoring System (MEMS), a pill-cap that contains an electronic micro-chip that records date and time of each bottle-opening. In contrast to other adherence measurement tools (such as self-reports, pill-counts, pharmacy refill data, and therapeutic drug monitoring), it combines objective data collection with detailed day-to-day information about medication intake over long time periods. MEMS-data have been found to accurately predict clinical outcomes in the treatment of HIV in a range of studies, and its use has been described in over 500 peer-reviewed publications [10,11].

Like the other adherence measurement tools, MEMS-caps data need to be evaluated for possible bias. Some suggest that MEMS-data may underestimate adherence due to suboptimal pill-bottle use (such as 'pocket dosing', i.e. removing multiple pills at once for later use) or overestimate adherence because of bottle-openings unrelated to medication intake [10]. Moreover, HIV-infected patients who fear the disclosure of their HIV-status have expressed concerns regarding the bulky design of the MEMS-bottle. This could affect consistent use of MEMS and the patients' willingness to participate in MEMS-research, especially in Sub-Saharan Africa where HIV-related stigma is highly prevalent [12]. Results from other studies suggest that monitoring ART adherence by using MEMS may improve medication intake, although the only study testing this experimentally revealed no such patterns [13,14]. In sum, electronic monitoring provides uniquely detailed and objective adherence data over long time periods, but the data-quality can be affected by the patients' acceptance and use of the device.

Although the importance of studying adherence to ART in developing countries is widely acknowledged, only a few studies report using MEMS-caps to monitor ART adherence in Sub-Saharan Africa [15,16]. This may be explained by the perception that MEMS-measurement is not feasible in these settings, or that MEMS-caps are too expensive to use in resource-limited settings. However, the costs of MEMS-caps seem modest (30 USD per 100 MEMS-caps for one year of data, 22 USD per 5,000 MEMS-caps for one year of data) when compared with the costs of clinical measures (e.g., 50 USD for a viral load measure and 25 USD for one CD4 cell count) [13]. Hence, with non-adherence preceding viral replication and thereby CD4 cell count decline as well as manifestation of clinical symptoms, electronic measurement of adherence could be a useful and affordable option in research and clinic practice in resource-limited settings.

Considering its potential usefulness for measuring adherence to ART, studies are needed that evaluate the feasibility of MEMS-monitoring in resource-limited settings. The aim of this study was to explore the patients' perceptions of and experiences with using MEMS-caps to monitor adherence in northern Tanzania.

## Methods

### Study site

The study was conducted in a rural ART clinic in the Kilimanjaro Region in northern Tanzania. The clinic provides services to about 700 PLWHA (of whom 70% are on ART) and is attended by 50 PLWHA on an average clinic day. There are two clinic days per week. Once in every two weeks on a clinic day, a health education session is conducted with all patients who are attending the clinic that day. The session takes about 15 minutes and is conducted by the clinician and a nurse at the clinic who provides practical information about adherence such as the importance of adherence and how to stay healthy in general. This health education session is usually followed by a discussion during which ART users ask questions and share their experiences in using ART.

### Inclusion criteria and study procedures

This study covered a three-months period during which participants were using their ART from MEMS-bottles, after which semi-structured interviews with the participants were conducted about their experiences with using MEMS-bottles. Ethical clearance was sought from the Tanzanian National Institute for Medical Research (NIMR) prior to commencement of the study (reference number: NIMR/HQ/R.8a/Vol.IX/812). Financial support for this study was obtained from the 'Innovation foundation health care insurers' (RVVZ, the Netherlands) and from the 'AIDS Foundation' (the Netherlands).

The eligibility criteria for participation in the study were: (1) aged 18 years or above; (2) being on ART for at least 6 months; (3) being a regular client to the clinic and not a temporary visitor; (4) being willing to participate in the study. All eligible patients who presented on one random clinic day were approached for participation. The researcher (author RL) was introduced to eligible patients by the ART clinician and explained that the aim of the study was to explore how patients perceive the use of a MEMS-bottle for their antiretroviral medication. Patients were informed that participation in the study was voluntary, that refusal would not affect their relationship with the healthcare provider, and that they were free to withdraw from the study at any time. Interested patients signed a consent form.

Study participants were informed that the MEMS-cap monitors pill intake behavior. The researcher instructed the patients as follows: (1) open the MEMS bottle only when taking medication and close it properly afterwards; (2) always take medication directly from the MEMS-bottle; (3) keep the MEMS bottle in a secured place out of reach of children or other people who may open the bottle; (4) take the MEMS-bottle along on clinic visits.

The study participants were followed for a period of three months (from mid-June to mid-September 2009). During this period, participants used their medication from the MEMS-bottle and took the MEMS-bottle along for medication refill during their monthly routine clinic visits. Only antiretroviral drugs were put in the MEMS bottles (no concomitant medication). At the clinic, patients handed over the MEMS-bottle to the data clerk who downloaded the data while the patients visited the clinician. Patients then picked-up the MEMS-bottle and proceeded to the dispensing nurse who filled the bottle with medication, after which patients returned home. Downloaded MEMS-data were not shared with study participants to avoid influencing their medication taking behavior, neither were they viewed by clinicians to avoid them acting upon the findings.

After three months, the patients were approached for qualitative, in-depth interviews. Patients were provided with a small compensation for the inconvenience of spending extra time at the clinic and to cover their transport costs.

### Data collection and analysis

A pre-tested interview guide with open-ended questions was used. The guide consisted of three major themes: (1) the MEMS design (opinions about color, shape and size); (2) the feasibility of using MEMS (ease of use, storage of the MEMS-bottle, portability of the bottle on travel occasions, opinions about the guidelines for correct use); (3) the impact of MEMS-use on adherence behavior and disclosure concerns.

All interviews were conducted in a private room at the ART clinic by three trained interviewers (authors RL, EM, and DM). Interviews lasted approximately one hour and were conducted in Kiswahili. All interviews were audio recorded and transcribed. Tape recordings and transcripts were used for data analysis, after which the tape recordings were destroyed as per ethical requirements. Transcribed data were coded independently by two researchers (RL and DM) and analyzed to identify common descriptive themes, which were then grouped into clusters.

Since self-reports on proper use of the MEMS-bottle may be biased by social desirability, misinterpretation of questions and responses, or memory retrieval problems, following the interviews we also examined the MEMS-data of all patients for: (a) patterns of overdosing (more openings on a day than prescribed) without MEMS-reports showing that patients ran out of medication before their next clinic visit (which should be the case if the overdoses are actual medication intakes and not curiosity openings); and (b) periods of >3 days during which the MEMS-cap registered no medication intake events, which could, apart from non-adherence, also indicate non-MEMS use. To distinguish between non-adherence and non-MEMS use, we first examined the interviews for patients reporting periods of non-MEMS-use. If patients had missing data but did not report inaccurate MEMS-use, we retrieved information on whether patients had collected their medication on time. If patients collected their medication too late in that period, the MEMS-data were considered accurate and the patient as non-adherent. If patients did not report inaccurate MEMS-use in the interview and were always on time to collect their medication, they were approached again and asked what happened in that period.

## Results

### Patient characteristics

All patients who attended the clinic on June 6^th ^2009 and met the eligibility criteria (n = 24), were approached for participation in the study and all agreed. However, one patient, a long-distance truck driver, withdrew from the study immediately after consenting because he judged the use of the MEMS-bottle to be incompatible with his travelling schedule. Hence, 23 ART users completed the three-month study period and were interviewed.

Fourteen (61%) participants were married, three (13%) were single, one (4%) was divorced, and five (22%) were widowed. All married participants had disclosed their HIV status to their spouses. The median age was 44 (interquartile range (IQR): 39-48). The period of ART use by the time of enrollment ranged from 6 to 48 months. At enrolment, the CD4 cell count was less than 200 cells/μL in one patient (4%), between 201 and 300 cells/μL in six patients (26%), between 301 and 500 cells/μL in ten patients (44%), and above 500 cells/μL in six patients (26%). The patient characteristics are summarized in Table 1.

**Table 1:** Demographic and disease characteristics of participants who completed the study (N = 23)

*Characteristics*	N (%)
Gender	
Female	14 (61%)

Marital status	
Married	14 (61%)
Single	3 (13%)
Divorced	1 (4%)
Widowed	5 (22%)

Months on ART	
6-12	2 (9%)
12-24	12 (52%)
24-48	9 (39%)

Education level	
No formal education	1 (4%)
Some primary education	4 (17%)
Completed primary education	15 (65%)
Secondary Education	3 (13%)

Occupation	
Subsistence farming	10 (44%)
Monthly Salaried work	4 (17%)
Days wage/Occasional work	5 (22%)
Petty business	4 (17%)

Distance to the clinic	
Less than 3km/walking distance	16 (70%)
Above 3 km	7 (30%)

CD4 cell count at baseline (cells/μL)	
>501	1 (4%)
301 - 500	6 (26%)
201 - 300	10 (43%)
<200	6 (26%)

### Acceptability of MEMS-design

The first indicator of the acceptability of the MEMS-bottle was the patients' willingness to participate in this study and use the MEMS-bottle. As reported, all eligible patients accepted study participation, but one withdrew from the study immediately after consenting. The majority of participants (21/23) indicated to have no problem with the color and size of the MEMS-bottle. A female participant (36 years) narrated: "*The shape is good; it looks like a make-up bottle*." Another female (41 years) stated: "*This container looks like a body lotion-it is not easy for people to know that I am carrying the pills*." A man of 52 years remarked: *"This medication bottle is better than ordinary pill bottles, its shape and size are OK." *Another man (30 years) said: *"If you put this bottle on the table, no one will know what it is for. Its size has no problem, and its color-no problem."*

Common feedback was that the MEMS-bottles, contrary to the regular medication bottles, do not have labels that reveal its content. This was perceived as highly beneficial. For example, a female of 39 years remarked: *"This container is very good. No one would immediately know what it is for. The normal pill bottles are labeled so when someone sees it, it is recognized quickly." *Another female (41 years) said: *"This one is cool. With the other type, people read it and start bombarding you with questions."*

Upon inquiring about what patients think the ideal shape of a MEMS-bottle would be, two participants proposed that a flat-shaped container would be easier to carry in one's pocket. A long-distance truck driver (male, 50 years) said: "*I would prefer a container with a flat shape like a small mobile phone because it will be easy to put in the pocket*."

### Feasibility of using MEMS according to guidelines

None of the patients reported to have had difficulties with following the guidelines for accurate MEMS-use: 1] to put the MEMS-cap on the pill bottle after use and close it properly, 2] always take all pills from the MEMS-bottle, and 3] prevent the bottle from being opened by a family member. Moreover, no one complained about the condition not to open the pill-bottle between medication intakes. A widower (44 years) said *"I keep it on my table, it's easy. I have my time for medication intake, so I open it during that time. On travel occasions, I move with it." *When asked if a family member or child might have opened the MEMS-bottle, most patients reported to have kept the MEMS-bottle out of reach from others. A woman (41 years) remarked: *"I did not have any problem whatsoever. I keep it in my bedroom and put it in a drawer to avoid it being opened by children. I have no problem with it, the instruction is not difficult." *One participant (male, 50 years) said: "*I hide my container in my suitcase. I don't want children or any other person to open it or ask me about it, this is my secret*."

Four participants talked about the challenges of always using all their medication from the MEMS-bottle. These challenges were mainly related to travelling. The patients considered it unsafe to travel with the bottle as they feared that it could be stolen or lost. For example, a long-distance truck driver (male, 50 years) indicated: *"When I was traveling, I left it at home and took the medication along. I was afraid the device could be stolen." *A female (39 years) confessed: "*I left my MEMS-bottle at home during my travel away from home and I transferred my pills into an ordinary medication bottle." *A mason (man, 50 years) said: "*I had to travel to Arusha for construction work for one and a half month. I left my MEMS-bottle at home and instead transferred my pills into another bottle. I feared my MEMS-bottle could be stolen or misplaced at the construction site."*

### Impact of MEMS-use on patient behavior

Nearly 75% of patients (17/23) had informed other family members that they had a new medication container. As one of the respondents said: *"I have informed my family and children that this bottle records pill taking behavior every time you open, so I strictly forbid them to touch it because it will be revealed when false openings are done to the pills bottle." *All participants who had told their family members about the MEMS-bottle said that their family members became more supportive in reminding them to take the medication. One participant (female 45 years) said: "*Because my family members don't want me to have a bad record on my medication, they are very supportive in reminding me about the time to take pills."*

On the question about how patients felt that their behavior was being monitored by the MEMS-cap, a typical response was: "*I have no problem with that." *A female (45 years) answered: *"That is very good because it motivates me to remember to take pills." *A man (30 years) remarked: *"I have become more conscious in taking medication. I am now more systematic and I don't forget to take my pills because I know that I am been monitored. With other containers I could take pills any time and skip taking pills at will." *Similarly, another man (45 years) said: *"Because I know that I am being recorded, I make every effort to take my medication as prescribed." *A women (45 years) stated: *"In the past I used to skip pills even for about 20 days, sometimes I found myself with two containers full of pills, but with this new container, I don't skip a day*."

### Corroborating interview responses with MEMS-data

Examination of the MEMS-reports revealed that there was no evidence of systematic overdosing by any of the patients. Bottles were typically opened twice a day at regular intervals. Sporadically, MEMS-bottles were opened three times a day instead of two. This was usually on clinic visit days when the bottle was refilled. Hence, these data confirm patients' reports that they only opened the pill-bottle for medication intake.

There were six patients whose MEMS-data showed periods of more than three days of missed medication (observed range was 12 to 71 days). Two of these patients reported during the initial interviews that they had left the MEMS-bottle at home when travelling, fearing that it might be stolen. Hence, they were not considered as non-adherent, but as not using the MEMS-cap. Two of the four remaining patients, who reported in the initial interviews to have always used the MEMS-cap as prescribed, showed up too late for collecting their medication in that period. Their missing MEMS-data was therefore considered to accurately describe their medication intake (i.e., non-adherence). Of the two remaining patients, who did not self-report inaccurate MEMS-use and were on time for collecting their medication, one could be re-contacted to explain the data. This patient reported to have left the MEMS-bottle at home when travelling for a period of six weeks. The non-monitored period was indeed 6 weeks, and this was thus considered to be non-MEMS-use rather than non-adherence. Hence, the MEMS-caps seemed to have been used accurately by all patients, except by three patients who did not use it during long-distance travels.

### MEMS-adherence scores

The interviews suggest that patients were, on average, highly adherent. Moreover, they suggest that patients changed their medication intake behavior because they were being monitored, an effect that has also been recognized in the Western literature. However, this monitoring effect is expected to reduce over time [16]. As a final step, we therefore explored the patients' monthly adherence scores, namely the percentage of doses taken (taking adherence) and the percentage of doses taken within a 9-15 hour interval (timing adherence), and explored whether adherence decreased over time also in this study. For the three patients who travelled and used their medication from another bottle, their period of non-MEMS-use was set to 'missing'. Monthly adherence scores were only computed for these three patients if they provided at least two weeks of continuous MEMS-data.

The adherence scores are summarized in Table 2 and reveal that adherence levels were fairly high and declined somewhat over time. Seventy-four percent (17/23) of the patients took more than 95% of their medication during the first month, 63% (14/22) in month 2 and 62% (13/21) in month 3. Wilcoxon's Signed Rank tests revealed that for taking and timing adherence there was a statistically significant decrease in adherence between month 1 and 3 (both p < 0.05), but not between month 1 and 2, or 2 and 3.

**Table 2:** Adherence scores per month

		1^st ^month (n = 23)	2^nd ^month (n = 22)	3^rd ^month (n = 21)
Taking adherence*	Mean (SD)	97.4 (4.7)	95.9 (5.78)	85.0 (26.8)
	
	Median (IQR)	98.2 (4.6)	96.4 (5.4)	98.2 (18.8)

**Timing adherence^**	Mean (SD)	94.2 (6.8)	92.6 (6.1)	82.1 (26.4)
	
	Median (IQR)	96.4 (4.6)	92.9 (6.3)	96.7 (20.5)

* Percentage of prescribed doses taken;

^ Percentage of doses taken within 12 ± 3 hours

One additional finding was that MEMS-data frequently revealed 3-5 days of non-adherence before a clinic visit. Upon exploring the cause of this pattern, it became clear that whereas the time between two consecutive visits in this clinic ranges from 30 to 35 days, patients were only provided with medication for 30 days. Hence, even the most motivated patients could not always be fully adherent due to mismanagement at the clinic level.

## Discussion

This study explored the feasibility and acceptability of using MEMS-bottles to monitor adherence to ART in Tanzania's Kilimanjaro Region. Twenty-three patients on ART used a MEMS-bottle during three consecutive months. After this period, in-depth interviews were conducted with the participants. Overall, patients perceived MEMS-bottles as simple and acceptable for use. The absence of a label on the bottle was perceived as an advantage to the ordinary pill-bottles and, unexpectedly, reduced fear for HIV-status disclosure. The majority of patients stated that the use of MEMS-caps had improved their adherence because of increased family support and the knowledge that the MEMS-bottle was monitoring their behavior. This is in line with findings by Wendel et al. [17].

Whereas we found that the MEMS-bottle was readily accepted by patients on ART, other studies found that some patients, especially those who prefer not to disclose their HIV-status, do not like the white, bulky appearance of the MEMS for privacy concerns [18-20]. The high acceptability of MEMS-use by patients in the current study is particularly remarkable because fears of disclosure of one's HIV-status and consecutive stigmatization are widely present in Tanzania and patients prefer to carry their medication as discretely as possible [21,22]. It turned out that the MEMS-design facilitated just that, with the absence of a medication label and the perception that the MEMS-bottle looks like a package of common products in this region, such as lotion or shampoo bottles.

The main barrier to using MEMS was on travel occasions. Three patients indicated to have left the MEMS bottle at home during travelling because of fears that it might be lost or stolen. This fear of losing the MEMS bottle on travel occasions was because patients perceived the MEMS-cap as a valuable device that could be stolen. This finding indicates the need of stressing patients that using the medication from the MEMS-caps when travelling is important and that the risk of it being stolen is acceptable and the responsibility of the researcher. This is especially important because adhering to the medication may be more challenging when travelling.

It has been suggested that MEMS monitoring can be perceived as intrusive or patronizing [20]. Patients in the present study perceived the monitoring of their adherence behavior as an advantage; they reported that their adherence had improved due to MEMS-monitoring. Most participants explicitly said that they paid more attention to taking their medication on the correct time because they were aware that their adherence behavior was monitored. Some patients admitted that they had been skipping medication intake for weeks in a row prior to starting to use the MEMS-bottle, but not anymore since MEMS-use. Adherence rates were indeed high, especially during the first month of monitoring. However, the levels of adherence tended to decrease after the first month of the study and then stabilized, suggesting that patients returned to their usual adherence behavior. This pattern has also been observed in other studies [23,24].

Examining the MEMS-data in order to determine periods of non-adherence or non-MEM-use, patterns of missed doses were found prior to clinic visits, because too few pills were being given to patients to bridge time between consecutive visits. Upon discussing the potential health consequences of this malpractice, the healthcare providers and dispensing nurse, acknowledging the necessity of a sufficient supply of medication, now provide patients with enough medication (including a buffer supply in case a patient is not able to visit the clinic on the appointed day).

This study showed that MEMS-devices have potential application in adherence research and monitoring of adherence in clinical practice in resource-limited settings. Although attention has to be paid to accurate MEMS-use, both at the time of instruction as well as at the moment of data interpretation, we did not observe some of the typical hurdles related to MEMS-use in resource-rich settings. For example, patients did not experience problems with the MEMS-design. In the absence of financial resources for regular viral load or CD4 testing, (intermittent) monitoring of adherence with the MEMS-cap may be possible and thus provide clinicians with valuable information to support the patients' medical treatment and medication adherence.

This study had some limitations. First, the sample size was small and the study was conducted in a single clinic, which limits external validity of the findings. However, the aim of the study was to explore the feasibility and acceptability of the MEMS-cap through qualitative procedures. Second, patients may not have understood well enough that their adherence data would not be shared with their doctor. The expectation that the doctor would act upon detection of non-adherence, might explain part of the intervention effect of MEMS-use, as described above. Third, the average adherence of patients with long periods of non-MEMS use should be interpreted with caution, since it cannot be verified whether medication was taken as prescribed in that period.

## Conclusions

This study suggests that the use of MEMS in a resource-limited, rural setting is feasible and acceptable to patients, and a potentially reliable source of information in settings where viral loads are not readily available. Special attention should be paid, though, to instructing patients about using the MEMS-cap while travelling. Moreover, researchers and clinicians are advised to develop procedures to verify whether missing MEMS-data was due to non-adherence or non-MEMS-use. Finally, although beneficial in clinical practice or adherence supporting interventions, the reported effects of MEMS-monitoring on adherence could introduce noise and threaten outcomes in research. Guaranteeing the patients' anonymity in data processing and analysis, and incorporating a period during which patients can get used to the MEMS-bottle are therefore advised.

## Competing interests

The authors declare that they have no competing interests.

## Authors' contributions

RL and DM contributed in study design, data analyses and interpretation, and drafting the manuscript. JB, EM assisted in data collection, transcribing and writing of the article. HH, AV and MdB contributed to study design, data interpretation and analyses, and drafting or reviewing the manuscript. All authors read and approved the final manuscript.

## Pre-publication history

The pre-publication history for this paper can be accessed here:

http://www.biomedcentral.com/1471-2458/11/92/prepub
